# Variations in growth, water consumption and economic benefit of transplanted cotton after winter wheat harvest subjected to different irrigation methods

**DOI:** 10.1038/s41598-019-51391-7

**Published:** 2019-10-18

**Authors:** Hao Zhang, Hao Liu, Shunsheng Wang, Xuan Guo, Lu Ge, Jingsheng Sun

**Affiliations:** 1 School of Water Conservancy, North China University of Water Resources and Electric Power, Zhengzhou, 450046 China; 2 Key Laboratory of Crop Water Use and Regulation, Ministry of Agriculture, P.R. China, Xinxiang, 453002 China

**Keywords:** Agroecology, Natural variation in plants

## Abstract

In the North China Plain (NCP), the utilization efficiency of cultivated land can be improved by transplanted cotton after winter wheat harvest (TCWWH). To understand the growth, water consumption and economic benefit of TCWWH under different irrigation methods, an irrigation experiment was carried out during 2013–2015 to explore the effects of border irrigation (BI), surface drip irrigation (SDI) and micro-sprinkling hose irrigation (MHI) on the plant development, water use efficiency (WUE) and economic benefit of TCWWH. The results showed that the survival rate of cotton seedlings in the SDI treatment was 12% and 7% larger than that in the BI and the MHI treatments, respectively. SDI increased plant height by 19% and 8% and increased leaf area index (LAI) by 24% and 17%, compared with BI and MHI, respectively. The highest seed cotton yield and better fibre quality were obtained in the SDI treatment, compared to the BI and the MHI treatments. Compared with BI and MHI, SDI reduced the soil evaporation and evapotranspiration (ET) in the field, and resulted in the largest WUE. The net profit generated by the SDI treatment exceeded that of the BI and the MHI treatments by 183% and 23%, respectively. Therefore, SDI can promote the growth of TCWWH and can increase the WUE and the economic benefit of TCWWH, compared with BI and MHI.

## Introduction

The Yellow River Basin in the North China Plain (NCP) is not only a primary cotton-growing region but also an important crop-growing region in general^1,2^. With economic development and rapid urbanization, cotton is competing with grain crops for cultivated land. A shortage of good-quality cultivated land and fresh water limits the rapid development of the agricultural economy in China, especially in the NCP^2^. Research shows that the transplanting technique combined with water-saving irrigation methods can improve the utilization efficiency of cultivated land and guarantee the harvest of both grain and cotton crops^2,3^. Compared with the traditional method of intercropping winter wheat and cotton, transplanting cotton after winter wheat harvest can improve the level of agricultural mechanization and increase winter wheat yield^4–6^. However, the transplanted cotton after winter wheat harvest (TCWWH) is short-season cotton, whose growth period in the NCP is mainly concentrated during the hot summer months (from June to September). While the average daily evapotranspiration (ET) in the TCWWH field is higher compared with the traditional direct-seeded cotton, the TCWWH yield is lower and the quality is worse^2,7^. The low yield and poor quality led to reduce economic benefit^2,8^. Thus, an optimal irrigation method must be selected to reduce the ET and improve the yield, quality and economic benefit of TCWWH.

Many researchers have reported that the growth, water consumption and economic benefit of cotton are significantly affected by irrigation methods^9–12^. In a study by Cetin and Bilgel^9^, sprinkler irrigation decreased water use efficiency (WUE) of cotton by 39%, while drip irrigation increased WUE by 26% compared to furrow irrigation (FI). Ibragimov *et al*.^11^ indicated that drip irrigation reduced the irrigation quota by 18–42% and increased the WUE of cotton by 35–103% compared to FI in Uzbekistan. Liu *et al*.^13^ found the average seasonal ET of transplanted cotton ranged from 358 to 449 mm, and the surface drip irrigation (SDI) reduced the irrigation quota by 33.4% compared with border irrigation (BI) in the NCP. Similar conclusions were summarized by Hodgson *et al*.^14^ and Aujla *et al*.^10^. Wang *et al*.^12^ indicated that the economic benefit of full irrigation reached its maximum value compared with that of medium and low irrigation in the northern Xinjiang of Northwest China. Wang *et al*.^15^ found that the single drip line design reduced the total input value by approximately 10% compared with the double drip line design, but the latter method produced more net income. Wang *et al*.^16^ compared mulched drip irrigation and flood irrigation to evaluate sustainable irrigation regimes for cotton in north western China. In addition, Lu *et al*.^17^ indicated that short-season cotton increased net profit by 69.2% compared with full-season cotton, as the material and labour input by the former was 27.3% less than that of the latter.

This study explored the effects of BI, SDI and micro-sprinkling hose irrigation (MHI) on the WUE and economic benefit of TCWWH. BI, a traditional irrigation method, is the most widely adopted method in the NCP^18^. SDI can be used for uniform and frequent water application in many soil and topographic conditions^9,19^. MHI is advantageous, and because of low investment cost, good anti-clogging performance and simple installation, it has been gradually adopted in the NCP in recent years^20^. Therefore, these three irrigation treatments (BI, SDI and MHI) were the focus of this study.

In this study, the main objective was to explore the optimal irrigation method for TCWWH from the perspective of WUE and economic benefit; at the same time, the effects of BI, SDI and MHI on WUE and economic benefit were investigated based on survival rate, plant growth, cotton yield, fibre quality, soil evaporation and soil water dynamics. The conclusions of this research are useful for popularizing the cropping pattern of TCWWH.

## Materials and Methods

### Experimental site

**Figure 1:**
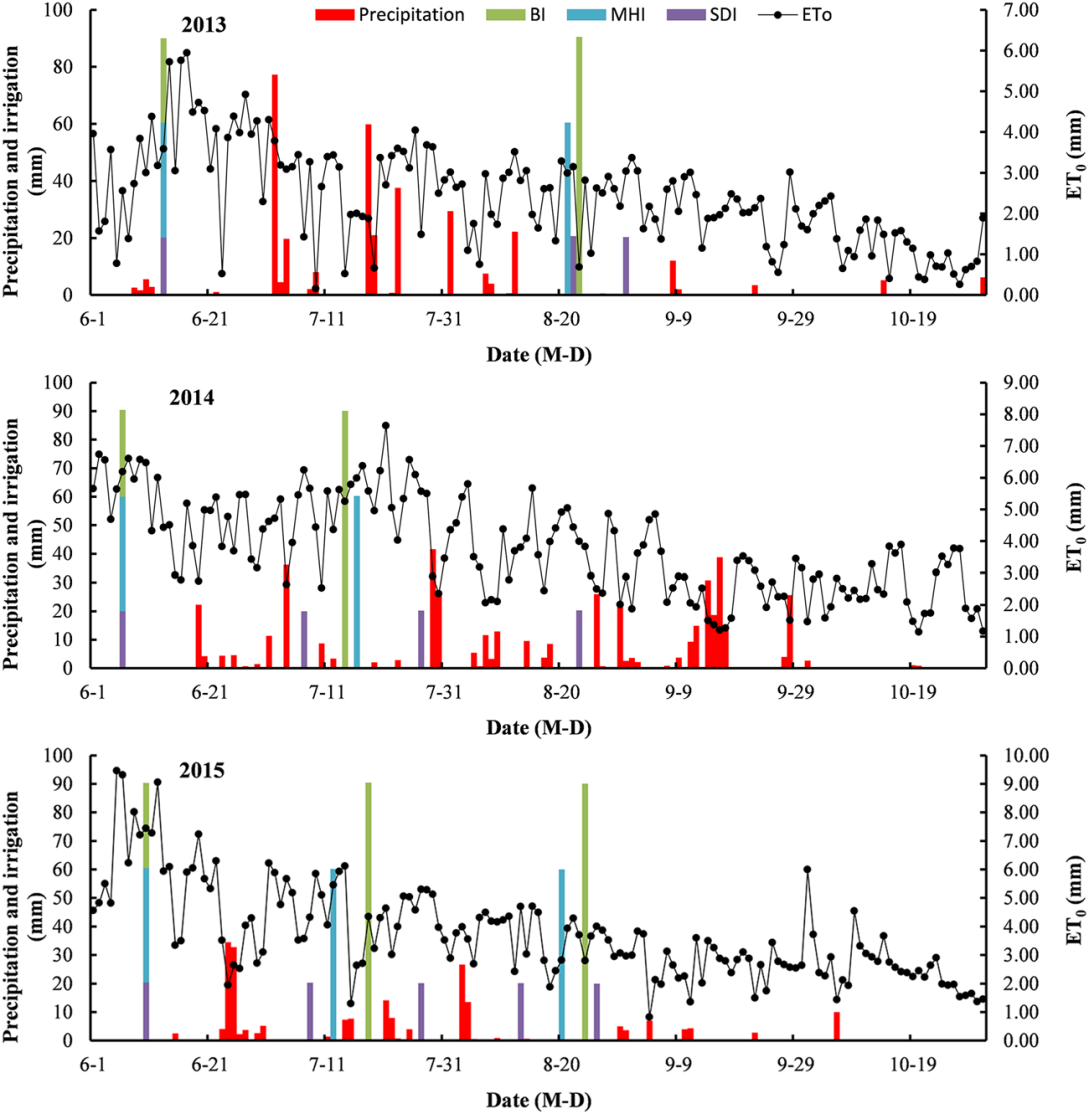
Precipitation, irrigation and *ET*_0_ during transplanted cotton after winter wheat harvest (TCWWH) seasons in 2013, 2014 and 2015.

This experiment was conducted from 2013 to 2015 at the Experimental Station of the Farmland Irrigation Research Institute, Chinese Academy of Agricultural Sciences (35°18′N, 113°54′E, altitude 73.2 m). The experimental site is located in a warm temperate climate region and has 220 frost-free days, a mean annual sunshine duration of 2286 h, a mean annual rainfall of 546 mm, and a mean annual temperature of 14.2 °C. The mean annual potential evaporation in the experimental site is 2000 mm, which was calculated using the Penman formulations^21^; the average groundwater table is below 5 m. The precipitation and *ET*_0_ are shown in Fig. 1, while the site’s physical and chemical soil properties are shown in Table 1.

**Table 1:** Soil physical and chemical properties in the experimental field.

Depth (m)	Soil texture	Particle size distribution (g 100 g^−1^)			Bulk density (Mg m^−3^)	Field capacity (m^3^ m^−3^)	pH	Available nitrogen (mg kg^−1^)	Available phosphorus (µg kg^−1^)	Available potassium (mg kg^−1^)	Organic carbon (g 100 g^−1^)
		<0.002 mm	0.02–0.002 mm	2.0–0.02 mm							
0–0.2	loam	4	43	53	1.56	0.34	8.9	59.3	12.0	146.1	1.3
0.2–0.4	silt loam	7	45	48	1.58	0.31	8.8	25.2	2.4	81.9	0.7
0.4–0.6	silt loam	6	48	46	1.54	0.33	8.8	18.6	1.9	77.0	0.8
0.6–0.8	silt loam	5	47	48	1.42	0.28	8.8	16.4	4.9	67.1	0.7
0.8–1.0	sandy loam	2	17	81	1.45	0.29	9.0	8.6	3.6	24.1	0.4

### Crop details

The Bt Cotton (Zhongmiansuo 50) was sown in separate cotton plug-seedlings on May 11, 2013; May 6, 2014 and May 6, 2015. The cotton seedlings were greenhouse-raised in substrate and, after approximately one month, were mechanically transplanted to the fields with a row spacing of 70 cm and interplant spacing of 20 cm. The planting density was approximately 71,400 plants ha^−1^. According to the locally recommended fertilizer practice, 450 kg ha^−1^ of compound fertilizer was applied to the soil as a basal fertilizer. After the squaring stage, 150 kg ha^−1^ of urea was applied. The harvest dates were October 20, 2013; October 13, 2014 and October 15, 2015.

Table 2 shows the growth schedule and the days per growth period of TCWWH. Since the TCWWH was short-season cotton, its growth time in the field was less than 130 d.

**Table 2:** Growth period of transplanted cotton after winter wheat harvest (TCWWH) in different years.

Year	Growth schedule (DAT)				Days per growth period (d)			
	Budding	Boll forming	Boll opening	Boll maturing	Seedling stage	Budding stage	Boll forming stage	Boll opening stage
2013	24	47	89	129	24	23	42	40
2014	24	49	91	129	24	25	42	38
2015	21	46	87	127	21	25	41	40

Five plants from the inner row of each treatment plot were randomly selected and labelled for the observation of plant reproductive development. When half of the labelled plants were budding, the date was recorded as budding time. The times of boll forming, opening and maturing were determined using the same method. Abbreviations: DAT, days after transplanting.

### Experimental design

Conducted through a completely randomized design, the experiment comprised of BI, SDI and MHI treatments; there were three repeated plots per treatment. Considering the marginal effect of different irrigation methods, the 9 plots were separated from adjacent plots by 4-m-wide isolation strips, and the size of each plot was 50.0 m long and 6 m wide. Table 3 shows the technical parameters for BI, SDI and MHI. After cotton seedlings were transplanted, we installed SDI and MHI systems in the field. The micro-sprinkling hoses were placed between two cotton rows, maintaining a spacing of 1.4 m in the MHI treatment. The drip pipe was laid on the cotton row, with each emitter placed beside cotton seedling.

**Table 3:** Technical parameters of the different irrigation methods.

Irrigation methods	Design specifications	Field surface slope	Irrigation flow	Equipment specifications
BI	Border width 2.1 m, border length 50 m, 3 rows of cotton in each plot	0.002	Flow per unit width into the furrow 4 L s^−1^ m^−1^	—
SDI	Laying length 25 m, tube spacing 0.7 m	—	Emitter flow 2.0 L h^−1^	Inter-tube dripper with 16-mm diameter, emitters were 20 cm apart, working pressure of 0.1 MPa
MHI	Laying length 50 m, hose spacing 1.4 m	—	Flow of 100 cm long hose 0.165 m^3^ h^−1^ m^−1^	Perforated hose with 40-mm diameter, 7 oblique water holes for one group in the hose, groups were 30 cm apart, working pressure of 0.4 MPa, spray width of 4 m

Once the soil water content (SWC) in the root zone of TCWWH was depleted to 70% of field capacity (F_C_), we applied irrigation for all treatments^11,22,23^. The irrigation quotas (90 mm for BI, 20 mm for SDI and 60 mm for MHI), which are shown in Fig. 1, were determined both by the practical experience of local farmers and were referenced from other studies^9,24,25^. We used a water meter to measure the amount of irrigation; the cotton seedlings were irrigated to guarantee their survival after transplantation.

### Measurement methods and calculations

Four cotton rows (10 m long) in each treatment plot were selected as the sample area to determine the survival rate (%) of the cotton seedlings. The survival rate was evaluated on the 21st day after transplantation and was calculated as follows:

1 $${\rm{Survival}}\,{\rm{rate}}=\frac{{\rm{Number}}\,{\rm{of}}\,{\rm{surviving}}\,{\rm{cotton}}\,{\rm{seedlings}}}{({\rm{planting}}\,{\rm{density}}\ast {\rm{sampling}}\,{\rm{area}})}$$

We randomly selected and labelled five cotton plants in each treatment plot to measure the plant height (cm) of TCWWH^7^. A ruler (accuracy of 0.1 cm) was used to measure the plant height at 7–10 d intervals from July to September^13^. A leaf area meter was used for the measurement of leaf area^26^, after which the leaf area index (LAI) was calculated by the FAO method^27^.

Daily soil evaporation (mm) was measured by micro-lysimeters in 2014 and 2015. The micro-lysimeter was made from galvanized iron and consisted of an inner and outer cylinder of 10 cm and 12 cm, respectively, and 15 cm in length. The outer cylinder was fixed into the soil, its top edge level with the soil surface to avoid disturbing the soil structure. The inner cylinder was pushed into soil until the top was level with the soil surface; its base was sealed with a plastic foil when it was removed. An electronic balance with a 0.1 g precision was used to weigh the inner cylinder daily at 8:00 AM. The soil evaporation is the difference between two adjacent measurements. The soil of inner cylinder was replaced every 2 d as well as after irrigation and rainfall to ensure accuracy of measurement. The micro-lysimeters were installed on the row and the middle of the inter-rows (35 cm from the row); six measurements were carried out for each treatment.

Time domain reflectometry with intelligent microelements (TRIME) was used to measure the SWC (cm^3^ cm^−3^) in the root zone of TCWWH at intervals of 20 cm from the soil surface to the maximum root depth every 3–5 d. The gravimetric measurement was employed to calibrate the measurement result^28^. There were six TRIME tubes in each treatment.

The boll number per labelled plant was recorded before the first harvest. Four cotton rows (10 m long) in each treatment plot were selected as the sample area to determine the seed cotton yield (kg ha^−1^) at every harvest, and the seed cotton yield was divided by the number of bolls to determine the boll mass (g). The lint percentage (%) was determined by ginning the seed cotton from each harvest^13^.

The fibre strength (g tex^−1^), fibre length (mm), micronaire, fibre elongation (%) and fibre uniformity (%) were determined by the high volume instrument system^7^.

ET (mm) was estimated as follows:

2 $${\rm{ET}}=P+I+U-{\Delta }S-{D}_{W}-R$$

where *P* is the precipitation (mm), *I* is the amount of irrigation (mm), *U* is the upward capillary flow into the root zone (mm), *ΔS* is the change in the amount of soil moisture storage (mm), *D*_*W*_ is the downward drainage out of the root zone (mm), and *R* is the runoff (mm). Due to the strict control irrigation during the growing seasons, *R* was never observed in the field. The *U* and *D*_*W*_ were calculated using Darcy’s law as follows:

3 $$q=-K({\psi }_{m})\frac{{\rm{d}}\psi }{{\rm{d}}x}$$

where *q* is the unsaturated and vertical one-dimensional soil moisture flux (mm d^−1^), *K* (*ψ*_*m*_) is the unsaturated hydraulic conductivity (mm d^−1^), $\frac{{\rm{d}}\psi }{{\rm{d}}x}$ is the total water potential gradient, *ψ* is the soil total water potential (cm), and *x* is the vertical distance (cm). *K* (*ψ*_*m*_) was measured by using the Ku-pF apparatus, while the soil matric potential was measured daily at 9:00 AM by using a tensiometer at soil depths of 90 cm and 110 cm^25,27,29^. The soil water characteristic curve was measured by using a high speed centrifuge, and RETC software was used to process the test data^30^.

The calculation for WUE (kg m^−3^) was as follows^13,29^:

4 $${\rm{WUE}}=\frac{Y}{10\times {\rm{ET}}}$$

where *Y* is the seed cotton yield (kg ha^−1^).

### Statistical analysis

To analyse the data obtained from the different irrigation treatments, the univariate GLM in SPSS Statistics 21.0 was used to make one-way analysis of variance (ANOVA) comparisons. The means were compared using least significant differences (LSD) at the 5% probability level.

## Results

### Survival rate and plant growth

Table 4 shows the survival rate of cotton seedlings in different irrigation treatments. SDI significantly increased the survival rate of cotton seedlings compared with BI and MHI. On average, SDI increased the survival rate by 12% and 7% compared with BI and MHI, respectively.

**Table 4:** Survival rate of cotton seedlings in different irrigation treatments.

Treatment	Survival rate (%)		
	2013	2014	2015
BI	86.0 ± 1.0 c	87.0 ± 2.6 b	87.7 ± 1.0 b
SDI	96.8 ± 1.2 a	97.8 ± 1.0 a	97.5 ± 1.0 a
MHI	90.0 ± 0.0 b	90.8 ± 3.8 b	90.0 ± 2.5 b

The deviations are the standard errors of the means. Mean values with different letters in the same column are significantly different at *P* < 0.05 level.

**Figure 2:**
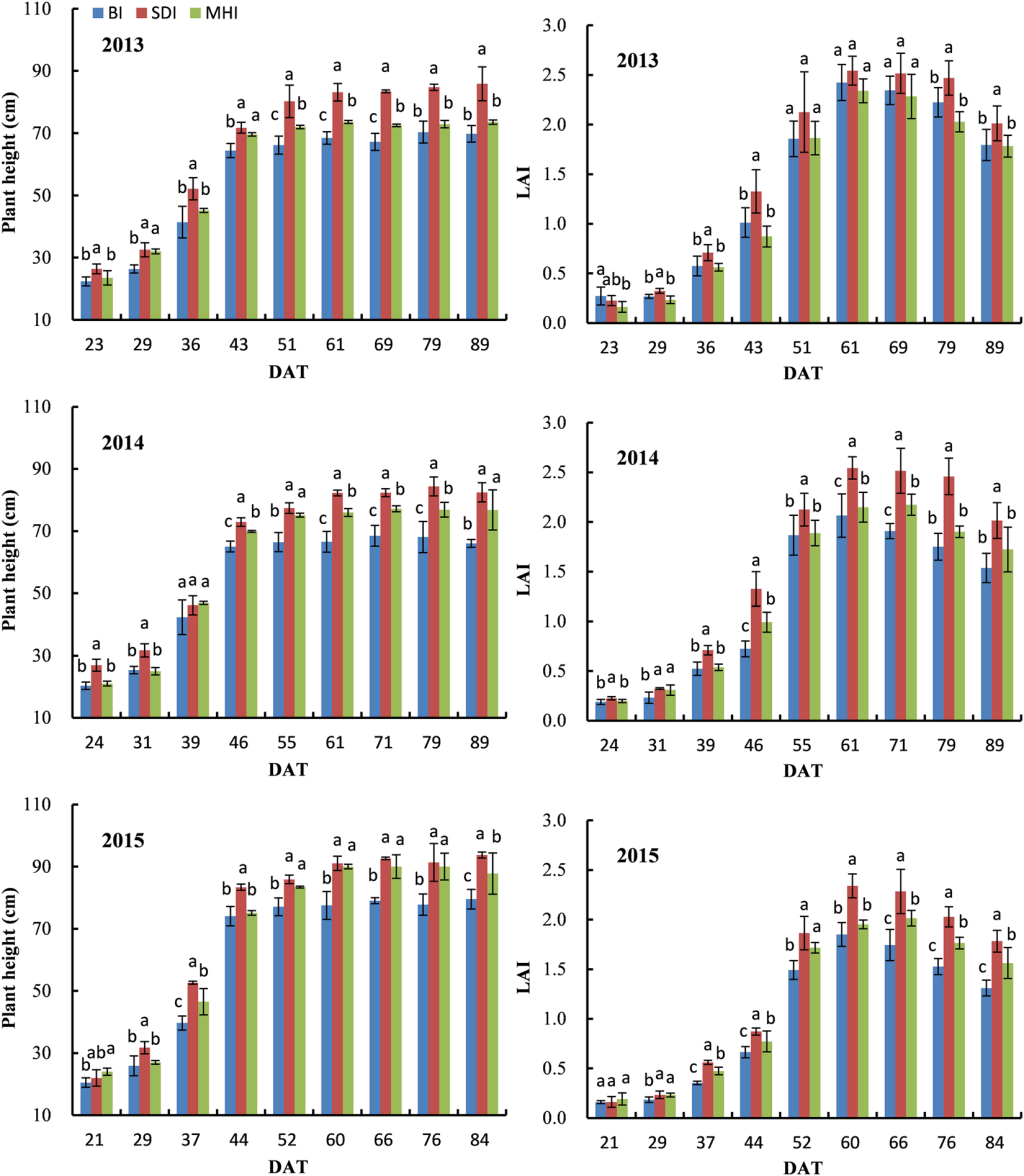
The variations in plant height and leaf area index (LAI) for different irrigation treatments. The vertical bars represent the standard errors of the means. Mean values with different letters are significantly different at *P* < 0.05 level. Abbreviations: DAT, days after transplanting.

The variations in plant height and LAI under different irrigation methods are presented Fig. 2. The changes in plant height and LAI in the seedling stage were small, while the changes in the budding stage were significant. At the beginning of the boll forming stage, the plant height of TCWWH reached its maximum of approximately 80 cm. The LAI of TCWWH reached its maximum (approximately 2.5) in the middle of the boll forming stage, and then gradually decreased. The plant height and LAI in the SDI treatment were the highest during the entire growth period, and lowest in the BI treatment. On average, SDI increased the plant height by 19% and 8% and increased LAI by 24% and 17% compared with BI and MHI, respectively.

### Cotton yield and fibre quality

Table 5 shows the seed cotton yield, lint yield, lint percentage, boll mass and number of bolls per plant. Compared with SDI and MHI, BI tended to decrease the boll mass and number of bolls per plant and to produce the lowest seed and lint cotton yields. BI significantly decreased the boll mass in 2013 and 2015, but the difference was not significant in 2014. Compared with BI, SDI increased the number of bolls per plant by 27% in 2014 and 18% in 2015 but decreased the number by 4% in 2013. The seed cotton yield of the SDI treatment was also the highest and ranged from 2683 (in 2014) to 3766 kg ha^−1^ (in 2015). On average, SDI increased the seed cotton yield by 32% and 9% compared with BI and MHI, respectively. The lint yield of the SDI treatment was also 32% and 10% higher than that of the BI and MHI treatments, respectively. For the lint percentage, the differences were not significant in the three experimental years.

**Table 5:** Cotton yield and yield components in different irrigation treatments.

Year	Treatment	Boll mass (g)	Number of bolls per plant	Seed cotton yield (kg ha^−1^)	Lint cotton yield (kg ha^−1^)	Lint percentage (%)
2013	BI	4.04 ± 0.15 b	9.1 ± 0.0 a	2617 ± 113 b	975 ± 37 b	37.3 ± 0.4 a
	SDI	4.41 ± 0.24 a	8.8 ± 0.1 b	3487 ± 132 a	1248 ± 40 a	35.8 ± 0.3 a
	MHI	4.54 ± 0.09 a	8.1 ± 0.2 c	3310 ± 35 a	1206 ± 21 a	36.4 ± 1.0 a
2014	BI	5.82 ± 0.12 a	6.5 ± 1.1 b	2142 ± 246 a	812 ± 112 a	37.9 ± 1.0 a
	SDI	5.86 ± 0.17 a	8.3 ± 0.6 a	2683 ± 136 a	1048 ± 82 a	39.0 ± 1.1 a
	MHI	5.93 ± 0.20 a	6.7 ± 0.3 b	2303 ± 350 a	872 ± 122 a	37.9 ± 1.2 a
2015	BI	3.84 ± 0.59 b	9.6 ± 1.3 b	2792 ± 128 b	1069 ± 41 b	38.3 ± 0.5 a
	SDI	6.39 ± 0.29 a	11.4 ± 0.2 a	3766 ± 91 a	1483 ± 46 a	39.4 ± 0.3 a
	MHI	5.43 ± 0.60 a	10.5 ± 0.6 ab	3516 ± 278 a	1359 ± 114 a	38.6 ± 0.4 a

The deviations are the standard errors of the means. Mean values with different letters in the same column are significantly different at *P* < 0.05 level.

Table 6 shows the quality parameters of cotton lint. SDI and MHI significantly increased the fibre length compared with BI. Over the three years, the average fibre length obtained in the SDI and the MHI treatments was 3.8% and 4.6% longer than that of the BI treatment, respectively. The fibre uniformity of the SDI and the MHI treatments was also higher than that of the BT treatment, but the differences were not significant. SDI and MHI significantly increased the fibre strength in 2013 and 2015 compared with BI but produced no significant differences in 2014. The effects of irrigation methods on micronaire and fibre elongation were too inconsistent to be definitively assessed. For all treatments, the average fibre strength, fibre length, fibre uniformity and fibre elongation of cotton lint in 2014 were generally lower than those in 2013 and 2015.

**Table 6:** Fibre quality parameters for different irrigation treatments.

Year	Treatment	Fibre length (mm)	Fibre uniformity (%)	Fibre strength (g tex^−1^)	Micronaire	Fibre elongation (%)
2013	BI	28.4 ± 0.1 c	83.3 ± 1.0 a	27.5 ± 0.4 b	4.5 ± 0.3 a	6.6 ± 0.1 a
	SDI	28.9 ± 0.2 b	83.9 ± 0.7 a	28.8 ± 0.3 a	4.4 ± 0.2 a	6.8 ± 0.2 a
	MHI	29.2 ± 0.2 a	83.7 ± 0.2 a	28.7 ± 0.2 a	4.6 ± 0.1 a	6.6 ± 0.1 a
2014	BI	25.7 ± 0.5 b	80.9 ± 1.6 a	27.9 ± 1.4 a	4.5 ± 0.1 a	6.3 ± 0.0 a
	SDI	27.3 ± 0.3 a	81.6 ± 0.9 a	27.0 ± 0.7 a	4.7 ± 0.1 a	6.2 ± 0.1 a
	MHI	27.6 ± 0.9 a	82.4 ± 0.7 a	27.8 ± 1.4 a	4.7 ± 0.3 a	6.3 ± 0.0 a
2015	BI	28.9 ± 0.2 b	83.6 ± 1.6 a	27.7 ± 0.3 b	4.5 ± 0.2 a	6.8 ± 0.0 b
	SDI	30.2 ± 0.4 a	85.3 ± 0.5 a	29.6 ± 1.3 a	4.5 ± 0.3 a	7.0 ± 0.1 a
	MHI	29.9 ± 0.2 a	85.3 ± 2.0 a	29.3 ± 0.4 a	4.5 ± 0.2 a	6.9 ± 0.1 b

### Soil evaporation and water dynamics

**Figure 3:**
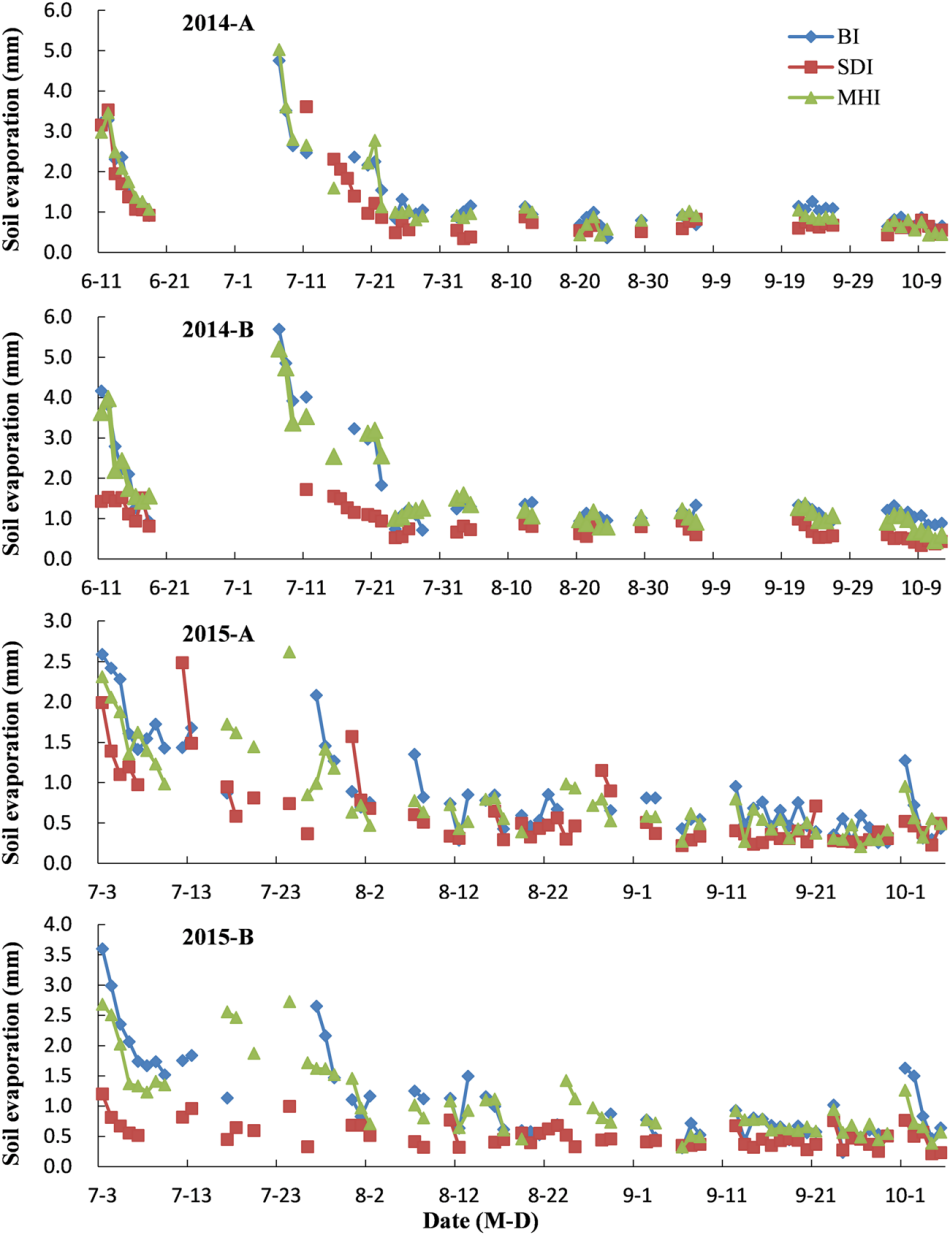
Diurnal variation in soil evaporation in the field of transplanted cotton after winter wheat harvest (TCWWH) in different irrigation methods. A and B represent different horizontal locations: A corresponds to the location on the row, and B corresponds to the location on the middle of the inter-rows.

Figure 3 shows the diurnal variation in soil evaporation in the different horizontal locations under the three irrigation methods in 2014 and 2015. The line interruption in Fig. 3 was due to the occurrence of rainfall or irrigation, which resulted in the failure of the data. Generally, the soil evaporation in the SDI treatment was the lowest and that in the BI treatment was the highest. Compared with BI and MHI, SDI decreased the soil evaporation on average by 28% and 23% in the cotton row (position A in Fig. 3) and by 51% and 50% in the middle of the inter-rows (position B in Fig. 3), respectively. In the SDI treatment, the soil evaporation at position A was 19% greater than at position B, while in the BI and the MHI treatments, the soil evaporation at position A was 20% and was 22% less than that at position B.

**Figure 4:**
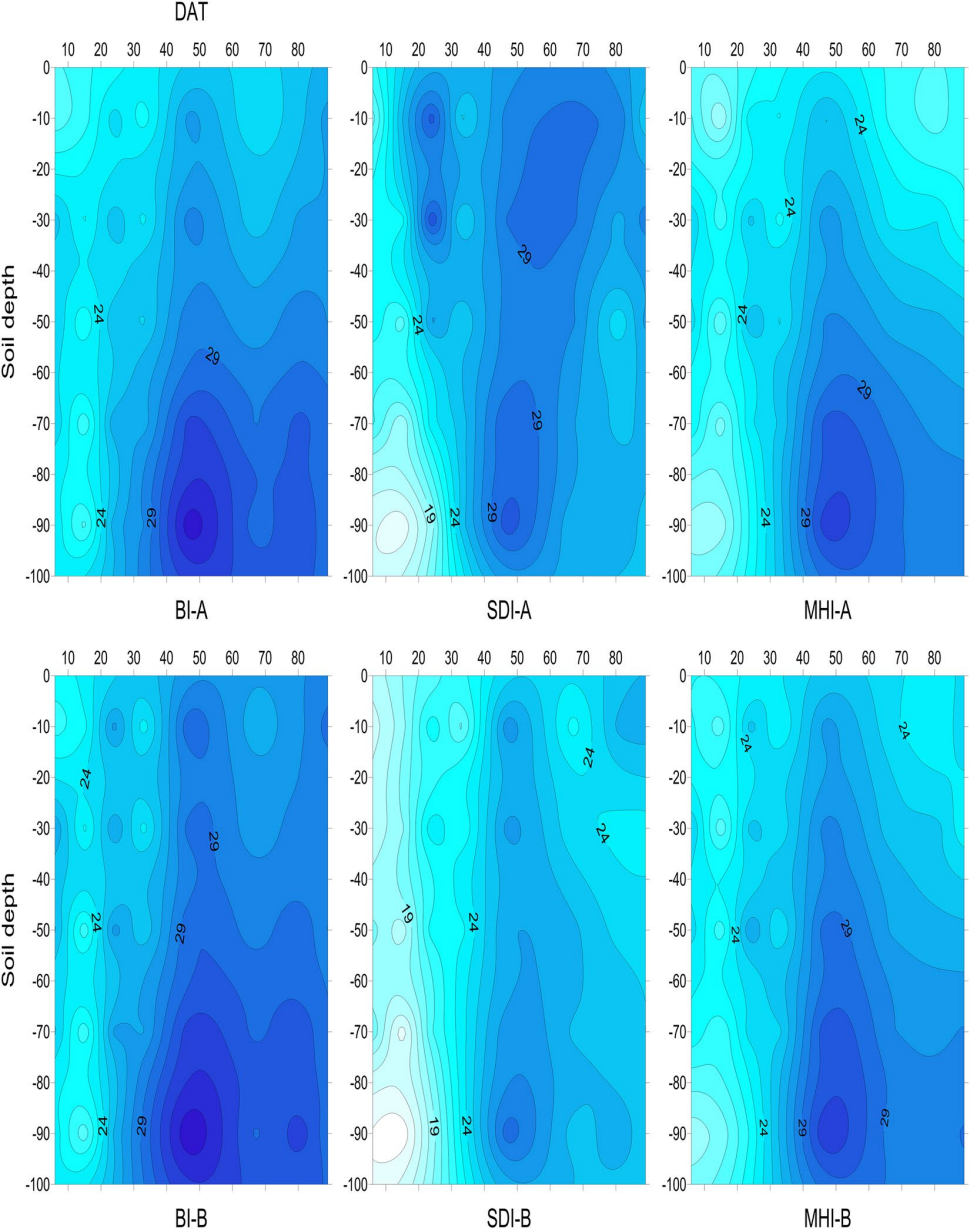
Dynamic change of soil water content (SWC, % cm^3^ cm^−3^) in the transplanted cotton field after winter wheat harvest (TCWWH) in different irrigation methods in 2013. A and B represent different horizontal locations: A corresponds to the location on the row, and B corresponds to the location on the middle of the inter-rows. Abbreviations: DAT, days after transplanting.

As similar tendencies for the SWC were observed in the three experimental years, only the 2013 data was selected to show the soil water dynamics under different irrigation methods. In the cotton plant row (Fig. 4A), the average SWC at depths of 0–40 cm in the SDI treatment was the highest and was 7% and 16% higher than that in the BI and the MHI treatments, respectively, in the same soil layer. The average SWC at depths of 40–100 cm in the SDI treatment was the lowest and was 10% and 4% lower than that in the BI and the MHI treatments, respectively, in the same soil layer. As shown in Fig. 4B, in the middle of the inter-rows, the average SWC in the SDI treatment was 16% and 10% lower than that in the BI and the MHI treatments, respectively.

### ET and WUE

The soil water balances of the 100 cm soil layer, ET and WUE in the field of TCWWH are summarized in Table 7. Under the same SWC limit (70% of F_C_) for different irrigation methods, the total irrigation quota in the BI treatment was 2.51 and 1.50 times larger than that in the SDI and BI treatments, respectively. Meanwhile, the D_W_ in the BI treatment was also the largest. SDI produced the lowest ET of 377.8 mm (in 2015) and significantly decreased the ET compared to BI and MHI. Over the three years, the average ET in the SDI treatment decreased by 19% and 10% compared to BI and MHI, respectively. The highest WUE of 1.00 kg·m^−3^ was produced in the SDI treatment (in 2015). On average, SDI increased the WUE by 62% and 21% compared to BI and MHI, respectively.

**Table 7:** Evapotranspiration (ET) and water use efficiency (WUE) of transplanted cotton after winter wheat harvest (TCWWH) in different irrigation methods.

Year	Treatment	*P* (mm)	*I* (mm)	*D*_*W*_ (mm)	*ΔS* (mm)	ET (mm)	WUE (kg m^−3^)
2013	BI	318.3	180.5	−2.0	−21.4	475.4 ± 20.0 a	0.55 ± 0.04 c
	SDI	318.3	81.3	0.0	−10.9	388.7 ± 3.2 c	0.90 ± 0.03 a
	MHI	318.3	120.9	0.0	−11.6	427.7 ± 3.6 b	0.77 ± 0.01 b
2014	BI	447.7	180.5	−59.3	−90.1	478.9 ± 5.1 a	0.45 ± 0.05 b
	SDI	447.7	80.5	−40.2	−108.0	379.6 ± 10.3 c	0.71 ± 0.03 a
	MHI	447.7	120.3	−50.9	−90.9	426.2 ± 1.9 b	0.54 ± 0.08 b
2015	BI	208.8	270.9	−22.8	0.3	457.3 ± 1.1 a	0.61 ± 0.03 c
	SDI	208.8	101.1	−9.1	77.0	377.8 ± 1.3 c	1.00 ± 0.03 a
	MHI	208.8	180.6	−2.6	38.6	425.5 ± 7.0 b	0.83 ± 0.07 b

Negative *D*_*W*_ values indicated the soil water seeped from the 100 cm soil layer into the deeper soil. Negative *ΔS* values indicated the SWC in the 100 cm soil layer increased compared to initial measurements, while positive *ΔS* values indicated the SWC decreased.

### Economic benefit

Table 8 shows the output, input value and net profit of TCWWH for different irrigation methods. On average, the output value generated by the SDI treatment was the highest, being 32% and 9% higher than that of the BI and the MHI treatments, respectively. However, SDI also increased the total input value, especially for the material cost, which became 36% and 8% higher than that of the BI and the MHI treatments, respectively. The net profit produced by the SDI treatment exceeded that of the BI and the MHI treatments by 183% and 23%, respectively. Compared to all three years, the net profit in 2014 was the lowest for all treatments.

**Table 8:** Economic benefit of transplanted cotton after winter wheat harvest (TCWWH) for different irrigation methods.

Year	Treatment	Output value ($ ha^−1^)	Input value ($ ha^−1^)			Net profit ($ ha^−1^)
			Material	Labour and machinery	Total	
2013	BI	2696.20	1058.96	1259.11	2318.07	378.12
	SDI	3591.73	1430.30	1086.48	2516.78	1074.95
	MHI	3410.18	1348.93	1110.63	2459.56	950.62
2014	BI	2068.21	1019.61	1040.61	2060.22	7.99
	SDI	2591.45	1389.98	799.17	2189.14	402.31
	MHI	2224.22	1282.29	823.31	2105.61	118.61
2015	BI	3145.33	1081.41	1354.48	2435.90	709.43
	SDI	4243.43	1483.17	1132.36	2615.53	1627.90
	MHI	3961.85	1343.38	1156.50	2499.88	1461.97

## Discussion

### Survival rate and plant growth

The survival rate of cotton seedlings affects the yield and economic benefit of TCWWH^31^. SDI significantly increased the survival rate compared to BI and MHI. This was because the roots of cotton seedlings were damaged while the cotton seedlings were transplanted in the field^3,4^, thus affecting their stability. The cotton seedlings were easily washed uprooted by the high velocity flow in BI and MHI, which led to their death. On the other hand, the water flow in the SDI treatment caused minimal damage to the cotton seedlings.

Suitable LAI and plant height are beneficial to ensuring an adequate canopy distribution and improving the utilization of solar energy, as well as the yield and quality of TCWWH^1,6,32^. The relationship between vegetative growth and yield of TCWWH is different from that of traditionally direct-seeded cotton. As the total growth time of TCWWH was approximately 45 d less than direct-seeded cotton, the plant height and the LAI of TCWWH were all smaller than those of direct-seeded cotton^2,4,5^. At the same time, many fine roots of cotton seedlings were damaged during field transplantation^3,4^, which led to the slow vegetative growth of TCWWH during the recovery stage. Poor vegetative growth resulted in the insufficient production of photosynthate, which reduced the yield and quality of cotton. SDI significantly increased the plant height and the leaf area of TCWWH compared with BI and MHI (Fig. 2), which was beneficial to the improvement of yield and quality of TCWWH. In the SDI treatment, soil water, heat, gases and the nutrients were best maintained and utilized for cotton growth, compared to the BI and the MHI treatments^7,33^; this is the main reason why SDI promoted the plant height and the LAI of TCWWH in this study. Irrigation methods could affect the micro-environment in the field, and the difference in atmospheric relative humidity within the canopy may also affect its growth^25^.

### Cotton yield and fibre quality

The yield and fibre quality of cotton are significantly influenced by irrigation^34^. In this study, the seed cotton yield of the SDI treatment was 23% larger than that of the BI treatment (Table 5). This was similar to the result reported by Rao *et al*.^35^, who reported the seed cotton yield of drip irrigation treatment was 33.5% larger than that in the BI treatment. However, in the study of Hodgson *et al*.^14^, SDI decreased seed cotton yield by 3% compared with FI. This was mainly due to the different limits of SWC between SDI and FI; the deficit below the fully-irrigated SWC in the FI treatment was maintained at 90 mm, whereas that in the SDI treatment was maintained at only 45 mm. The seed cotton yields reported by Hu *et al*.^22^ and Hu *et al*.^22^ were higher than those in this study (Table 5), potentially due to differences in growth time and cotton varieties. This study used the short-season cotton variety whose total growth time was less than 160 d. Lu *et al*.^17^ indicated that short-season cotton decreased the seed cotton yield by 14.5% compared to full-season cotton. Cotton yield was also affected by agricultural practices and climate in the different regions. For all treatments, the seed yield of TCWWH as well as the fibre strength, fibre length, fibre uniformity and fibre elongation of cotton lint in 2014 were all lower than those in 2013 and 2015 (Tables 5 and 6). This may be due to the continuous rainfall during the mature and harvest stage in 2014 (Fig. 1).

### Soil evaporation and water dynamics

As only the cotton row was irrigated and the irrigation amount was sufficiently low (20 mm) in the SDI treatment, the SWC on the inter-rows or in deep soil depths was lower than that in the BI and the MHI treatments. This distribution characteristics of SWC in the SDI treatment reduced soil evaporation and prevented deep infiltration, reducing the loss of water and nutrients^36^. The soil moisture distribution in the SDI treatment was the most favourable for cotton root growth compared with BI and MHI^22,37^; the developed root then promoted the growth of the cotton canopy. This is also the reason why SDI promoted the yield and fibre quality of TCWWH.

### ET and WUE

As freshwater shortage limits the sustainable development of agriculture in the NCP^38,39^, it is very important to study the water-saving irrigation methods for TCWWH. SDI significantly decreased the ET compared with BI and MHI (Table 7). Rao *et al*.^35^ reported a similar result of water in the drip irrigation treatment to be 30% less than that in the BI treatment. SDI increased the WUE compared with both BI and MHI (Table 7), which was similar to the results observed by Cetin and Bilgel^9^ and Ibragimov *et al*.^11^. The WUE in the SDI treatment in this study varied from 0.71 to 1.00 kg m^−3^; this was higher than the results reported by Cetin and Bilgel^9^ and Yazar *et al*.^40^. Although a greater seed cotton yield was obtained, more water was consumed and lower WUEs were obtained in their studies.

### Economic benefit

In the Yellow River Basin of the NCP, farmers have gradually abandoned the traditional method of intercropping winter wheat and cotton and are adopting the model of TCWWH^2^. In this region, the industrialized production of cotton seedlings has begun to emerge, and farmers widely use machinery to complete agricultural activities such as winter wheat harvesting, land ploughing and cotton seedlings transplantation. TCWWH is more conducive to mechanization and can reduce labour expenditure, compared with cotton intercropped with winter wheat^2,4–6^. Lu *et al*.^8^ indicated that transplanted cotton increased net profit by 10.9% and 31.8% in the low and high fertility field, respectively, compared with mono cropped cotton. The net profit of TCWWH produced by the SDI treatment was the highest compared to that of the BI and the MHI treatments, even though the total input value for the SDI treatment was also the highest (Table 8). The increased profit produced by the SDI treatment was mainly attributed to the high seed cotton yield (Table 5). In 2014, the seed cotton yield was very low due to the influence of continuous rainfall during the boll maturing stage of TCWWH (Fig. 1), and the net profit this year was also the lowest. SDI and MHI consumed more material input but less labour and machinery input than BI due to the very high depreciation cost of irrigation equipment in the SDI and the MHI treatments, while BI needed additional labour and machinery input for irrigation management and land leveling^12,15,17^.

## Conclusions

This study showed that SDI can increase the WUE and economic benefit of TCWWH compared with BI and MHI. The survival rate of cotton seedlings in the SDI treatment was larger than that in the BI and the MHI treatments. Compared with BI and MHI, SDI improved the plant height, LAI and yield of TCWWH and decreased the soil evaporation and ET in the field; it also improved the fibre quality compared to the other treatments. Therefore, SDI is the optimal irrigation method for the TCWWH in the NCP.
